# Sequencing rare T-cell populations

**DOI:** 10.18632/oncotarget.6349

**Published:** 2015-11-18

**Authors:** Mikhail Shugay, Sergey Lukyanov, Dmitriy M. Chudakov

**Affiliations:** Shemyakin_ovchinnikov Institute of bioorganic chemistry RAS, Miklukho-Maklaya, Moscow, Russia and Pirogov Russain National Research Medical University, Ostrovityanova, Moscow and Central European Institute of Technology, Masaryk University, Brno, Czech republic

**Keywords:** TCR profiling, unique molecular barcodes, minor lymphocyte subsets

High-throughput sequencing of T cell (TCR) receptor repertoires is a promising approach that can be used to characterize the state and dynamics of adaptive immunity and, potentially, to deduce the antigen specificity of the immune response [1].

While the number of applications of TCR profiling is constantly growing, basic protocols have several drawbacks that are making it difficult to utilize them in certain experimental settings. In particular, quantitative data interpretation is hampered by stochasticity of sampling of lymphocytes and TCR-encoding RNA/ DNA, stochasticity and biases of PCR amplification, and sequencing quality biases.

These problems become more evident when profiling rare T-cell subpopulations, such as tumor-infiltrating lymphocytes (TILs) that may contain low lymphocyte counts, populations enriched for tumor antigen-specific cells, or functional T cell subests of interest, the tasks critical for studying the role of adaptive immunity in cancer progression and treatment.

In this brief note we summarize what we suggest to be the most relevant issues of studying tumor-specific TCR repertoire for the minor lymphocyte counts, where derived data is more prone to noise introduced by stochastic sampling, which renders methods that operate with T-cell clonotype frequencies less useful.

**Figure 1:**
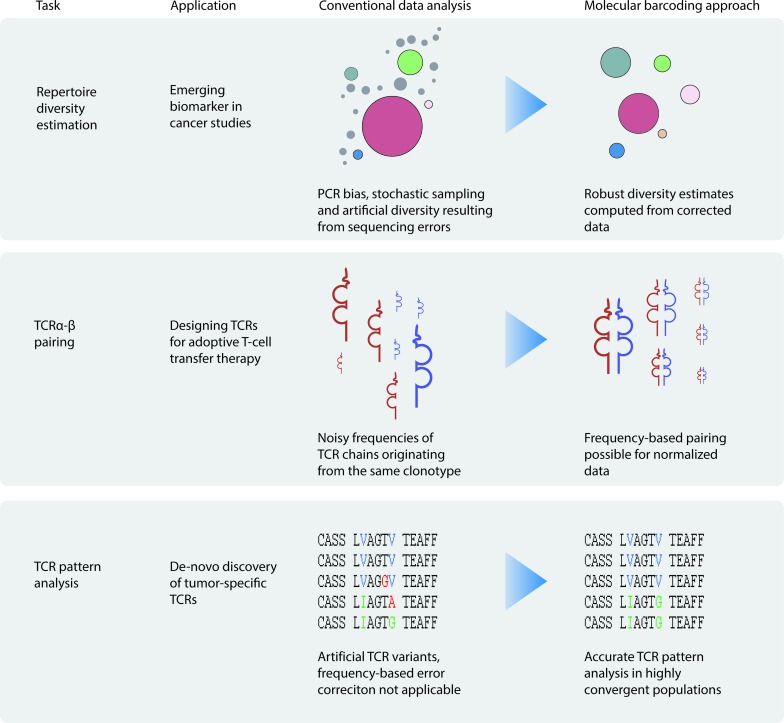
Common analysis tasks applicable to repertoire sequencing of tumor infiltrating lymphocyte populations and sorted T-cells specific to tumor antigens.

As a way to resolve these challenges we refer to our recently published highly-sensitive T-cell repertoire profiling protocol that enables studying rare lymphocyte populations [2]. The protocol employs molecular barcoding technique that was proven to be extremely useful for the immune repertoire profiling task [3-5]. This approach allows tracing raw sequencing reads to their initial cDNA molecules, yielding robust immune repertoire data that is normalized and corrected for PCR and sequencing errors (Figure 1).

First, molecular barcoding provides control for the experimental bottlenecks. This is critical when working with limited available material, as researcher is blind in the respect of the counts of analyzed cells and molecules when using conventional approaches. For example, 10 molecules that were sufficiently amplified and analyzed with 100,000 sequencing reads may look like a highly convergent “repertoire” after all accumulated errors. Molecular barcoding provides a mean to analyze immune repertoires for the known number of starting TCR cDNA molecules, which closely reflects the count of the analyzed lymphocyte population [5].

Second, both normalization to the defined numbers of analyzed cDNA events and elimination of PCR and sequencing error and artifacts are important for calculating robust TIL repertoire diversity estimates [6], an emerging biomarker shown to possess a high prognostic value in cancer setting [7].

Third, molecular barcoding drastically improves quantification of clonotypes, and thus allows to perform frequency-based pairing of TCR alpha and beta chains for the largest clonotypes. Knowing functional alpha-beta pairs is critical for designing transgenic TCRs for adoptive T-cell therapy [8].

Finally, highly homologous TCR variants may be represented by major and minor clonotypes, the latter being indistinguishable from the accumulated PCR and sequencing errors using conventional algorithms. At the same time, antigen-specific T-cell subpopulations, e.g. sorted using MHC-peptide multimers, as well as tumor-infiltrating clonotypes that are specific for particular tumor antigens, may include convergent TCR variants [9]. Molecular barcoding so far is the only approach that can correct for PCR and sequencing errors in highly convergent populations without losing information on original repertoire diversity [4].

With optimized solutions being readily available for sequencing rare T-cell populations, we expect for a boost in TIL profiling studies that will provide data for rational development of cancer immunotherapies, as well as identification of antigen-specific TCR variants aid in developing new strategies in the field of adoptive T-cell therapy. Ultimately, these studies will bolster our understanding of the mechanisms behind T-cell response in clinical setting with an index of TCR variants of known antigen specificity.
